# Livestock sustainability research in Africa with a focus on the environment

**DOI:** 10.1093/af/vfab034

**Published:** 2021-09-06

**Authors:** Mulubrhan Balehegn, Ermias Kebreab, Adugna Tolera, Sarah Hunt, Polly Erickson, Todd A Crane, Adegbola T Adesogan

**Affiliations:** 1 Feed the Future Innovation Lab for Livestock Systems, University of Florida, Gainesville, FL; 2 Department of Animal, Rangeland and Wildlife Sciences, Mekelle University, Mekelle, Tigray, Ethiopia; 3 Department of Animal Science, University of California Davis, Davis, CA; 4 Hawassa University, Hawassa, Ethiopia; 5 International Livestock Research Institute, Nairobi, Kenya; 6 Department of Animal Sciences and Food Systems Institute, University of Florida, Gainesville, FL

**Keywords:** enteric methane emission, silvopasture, sub-Saharan Africa, sustainable intensification

**Implications**

- Livestock in African countries contribute to about 10% of enteric methane emissions from dairy cattle worldwide despite producing only 3.9% of the world’s milk.
- Livestock in Sub-Saharan Africa also cause extensive land degradation with 48% of rangelands in SSA degraded due to overgrazing.
- Strategies for sustainable intensification of livestock such as improving quality of feed, range and grazing land rehabilitation, introduction of improved forages and silvopastoral systems, and improvement of herd genetics can reduce both total emission and emission intensity while improving productivity.
- Sustainable intensification strategies are not always readily adopted, therefore, smallholder farmers in Africa require policy directives, financial and technical support.

## Introduction

Africa’s livestock accounts for one-third of the global livestock population (AU-IBAR, 2016) and about 40% of agricultural GDP in Africa, ranging from 10% to 80% in individual countries (Panel, 2020). Livestock will be increasingly important in the future in sub-Saharan Africa (SSA) because the demand for animal-source food (ASF) is projected to increase due to population growth, increased incomes, and urbanization. By 2050, consumers in low- and middle-income countries will demand 107 million tons more meat and 5.5 million tons more milk than they did in 2005/2007 (Alexandratos and Bruinsma, 2012). The current per capita annual consumption of meat and milk of about 14 kg and 30 L, are projected to rise to 26 kg and 64 L, respectively, by 2050 in SSA (AU-IBAR, 2016). However, African livestock also have significant impacts on the environment (Gerber et al., 2013). More than 70% of agricultural GHG emission in Africa comes from the livestock sector dominated by enteric methane (CH_4_) emission (Tubiello et al., 2014). Greenhouse gas emission intensity (i.e., emissions per unit of livestock product) in east Africa is four times greater than the global average (Pressman et al., 2018), and estimates from other parts of SSA are likely similarly high. The North American average milk yield of 9,000 kg/cow per year is much greater whereas the carbon footprint of 1.3 kg of CO_2_-eq./kg of milk is much lower than the corresponding SSA estimates of 250 kg/cow per year and 7.6 kg of CO_2_-eq./kg of milk, respectively (Capper, 2011). Livestock production in SSA causes extensive land degradation, overgrazing, and associated loss of biodiversity. For instance, UNEP (1992) reported that 58% of all land degradation and soil erosion in SSA is caused by overgrazing. This is because as the human population increases, more and more pastureland is used for food crop cultivation, increasing stocking rate, and grazing pressure on remaining grazing lands. A study in central Ethiopian highlands (Mekuria et al., 2018) showed that cropland increased by 16% while grazing lands decreased by 52% from 1984 to 2016. Free grazing livestock also hamper soil and water conservation in degraded areas (Giday et al., 2018). If agricultural knowledge, science, and technology do not improve, a general decline in species abundance in Africa due to livestock grazing is predicted at least until 2030 (Alkemade et al., 2013). Livestock grazing also causes encroachment on rangelands of grazing-resistant and unpalatable bushes, severely reducing rangeland plant biodiversity (O’Connor et al., 2014). This review examines the state of knowledge, policies, and practices on sustainability of livestock production in Africa and changes needed to drive improvements.

## Sustainability of Livestock Production in Africa

In Africa, sustainable livestock production must address food security and climate change concerns simultaneously in addition to social and economic aspects. The need for and principles of sustainable livestock production apply universally. Although many high-income countries focus on the environmental impacts of livestock production, low-income countries are concerned with increasing livestock productivity to improve income and food supply and reduce high rates of childhood undernutrition and stunting (Tricarico et al., 2020). Currently, most countries in Africa rely on the Intergovernmental Panel on Climate Change (IPCC) tier 1 methodology to estimate their livestock-based emissions. However, detailed, and precise activity data are lacking, and accurate estimates of natural resource use and environmental impact by livestock in Africa, particularly SSA are scarce.

## Greenhouse Gas Emissions from African Livestock Systems

**Figure 1.**
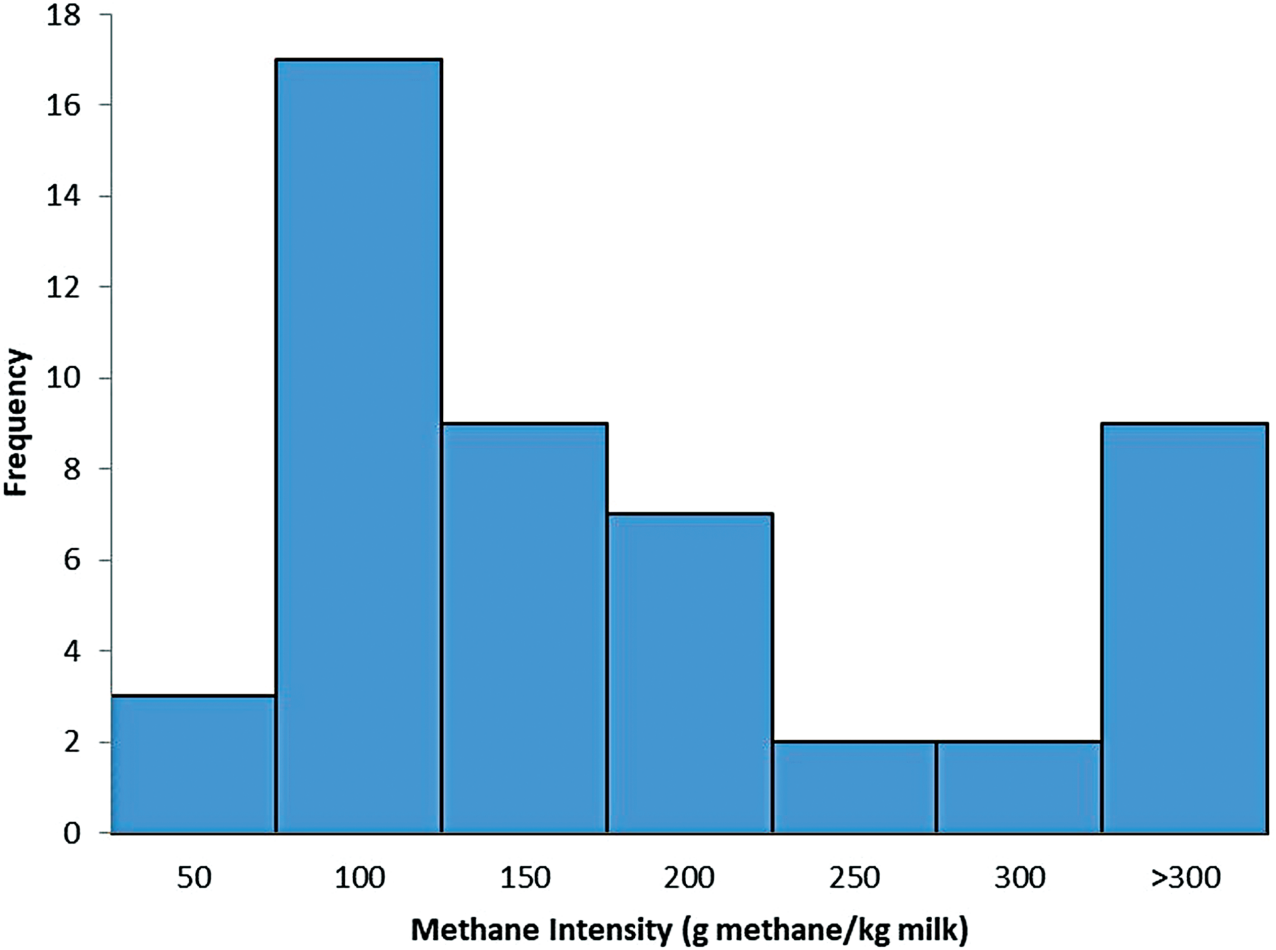
A histogram showing the number of African countries that have a CH_4_ intensity (g CH_4_/kg milk) factor within each class (based on FAOSTAT (2018) database).

Global greenhouse gas emissions from livestock were estimated at 7.1 Gt CO_2_-eq./annum in 2013, which is 14.5% of human-induced GHG emissions (Gerber et al., 2013). Enteric fermentation from ruminants is only second to feed production in its contribution to overall GHG emissions from livestock. Gerber et al. (2013) reported that ruminants in SSA contribute a large share of global GHG emissions due to their high emission intensity. This is because of the low productivity levels of African livestock. For instance, the mean milk production in Africa ranged from 108 to 3,368 kg/cow per year (IPCC, 2019), with about half of the countries producing below 500 kg milk/cow per year (FAOSTAT, 2018). Consequently, African countries contribute to about 10% of enteric CH_4_ emissions from dairy cattle worldwide despite producing only 3.9% of the world’s milk (FAOSTAT, 2018). Therefore, the intensity of CH_4_ emissions (g/kg milk) is much larger in Africa compared to the rest of the world. For comparison, in the United States, the average CH_4_ intensity is about 13 g CH_4_/kg milk. Figure 1 shows an analysis of the FAOSTAT (2018) database indicating that most African countries had intensity values of 50 to 100 g CH_4_/kg milk with nine countries over 300 g CH_4_/kg milk.

Regional analysis of CH_4_ intensity in Africa showed that the systems with the lowest values (most efficient) were in Northern Africa (mean = 46 ± 12 g CH_4_/kg milk). In these countries, the dairy production system relies mainly on irrigated forages and uses either high genetic merit or crossbred cows (Sraïri et al., 2013). Northern African farmers use off-farm high-quality purchased feed resources which may represent up to 60% of the total energy intake (Kadi and Djellal, 2009). However, water is a major constraint to increasing forage production in this region. Sustainability of farms in the region relies on technologies that improve water use efficiency for feed production (Sraïri et al., 2013).

Southern Africa had the second lowest CH_4_ intensity (mean = 102 g CH_4_/kg milk: SD = 56 g CH_4_/kg milk). The CH_4_ intensity range in this region is greater compared to Northern Africa covering 25.5 (South Africa) to 226 g CH_4_/kg milk (Madagascar) partly because many of their dairy farms have more than 500 cows and use a total mixed ration (TMR) feeding system leading to annual milk production of over 3,000 kg/cow (Dolecheck and Bewley, 2018). Whereas, in countries such as Madagascar and Zimbabwe, annual milk production is <500 kg/cow leading to much greater CH_4_ intensity.

Western Africa had the third lowest CH_4_ intensity ranging from 54.3 g CH_4_/kg milk in the Republic of Congo to 610 g CH_4_/kg milk in Côte d’Ivoire (mean = 194 g CH_4_/kg milk; SD = 176 g CH_4_/kg milk). The wide range in CH_4_ intensity in the region reflects the various production systems and efficiency of milk production. About 50% of milk in Western Africa is produced by nomadic herders and agro-pastoralists (Seyoum, 1988) that use local breeds with low-quality feeds, causing greater CH_4_ intensity.

The greatest CH_4_ intensity was in Eastern Africa with an average of 233 g CH_4_/kg milk, but the intraregional variation was smaller compared to Western Africa (SD=110 g CH_4_/kg milk). This region is characterized by mainly smallholder farmers, who experience various challenges including problems with overgrazing, a lack of infrastructure (very few milk collection locations and product manufacturers), little access to credit, and difficulty transferring technical knowledge and skills to farmers (Dolecheck and Bewley, 2021).

The CH_4_ intensity of beef production in Africa is also high, particularly in SSA. The enteric CH_4_ emissions values were based on IPCC (2019) tier 1 calculations because of lack of research that directly measures emissions. A recent study using respiration chambers showed that that tier 1 emission factors from IPCC overestimated both CH_4_ and nitrous oxide emissions from cattle excreta in Kenya, given typical smallholder practices in Eastern Africa (Pelster et al., 2016). The Feed the Future Innovation Lab for Livestock Systems is addressing this problem by installing state-of-the-art CH_4_ measurement devices (GreenFeed, C-Lock Inc., Rapid City, SD) for large and small ruminants in East and West Africa, respectively, to quantitatively measure CH_4_ emissions from tropical cattle. Data from this type of research will lead to better quantification and development of the tier 2 system that takes into account factors that affect emissions such as diet quality and feed intake.

## Technologies to Improve Sustainability of Livestock Production in Africa

### Improving quality of feeds

Most ruminant feeds in Africa are low-quality crop residues and natural pasture making up 72% to 93% of total feed consumed by livestock (FAO, 2014). Improving crop residue entails increasing the crude protein concentration and/or digestibility using physical, biological, or chemical treatment of crop residues, thus potentially increasing intake and digestibility and reducing enteric CH_4_ emission intensity. This approach is also more feasible than concentrate supplementation due to the lower cost. Ammonization and alkali treatment of roughages reduces methane intensity (i.e., decrease methane produced/unit of output) by increasing animal productivity. However, urea treatment is more commonly used than alternatives and has the greatest uptake potential when the costs, hazards, and efficacy of chemical treatments are considered (Woyengo et al., 2004). Using urea-treated crop residues in ruminant diets was compared with traditional diets (untreated straw) and resulted in reduced GHG emissions by 1.02 Mt CO_2_-eq. annually (5.5%) in Ethiopia and 3.18 Mt CO_2_-eq. annually (8.8%) in Kenya (Pressman et al., 2018). The adoption of crop residue improvement technologies in Africa, however, is limited by the lack of technologies that fit the social, economic, and ecological conditions. Research is, therefore, required to develop technologies that are fit to smallholder production systems (Owen et al., 2012).

Physical crop residue treatment techniques such as grinding, chaffing, and chopping of roughage or fibrous feeds also have reduced enteric CH_4_ emission by about 10% through improvement of feed intake, digestibility, and improved microbial activity or fermentation as a result of increased surface area of the substrate (Valli, 2020). Similarly, pelleting reduces feed waste by up to 5%, thus reducing emissions, while reduction in particle size from 1,000 to 600 microns increases dry matter and nitrogen digestibility by 5% to 12% and reduce nitrogen in manure by 20% to 24% (Carter et al., 2012).

Quality of crop residues can also be improved through crop breeding particularly when aimed at improving the digestibility of dual-purpose crops. Crop residues or other fibrous feeds differ in their digestibility and, therefore, enteric methane emission (Giger-Reverdin and Sauvant, 2000). A cultivar-dependent difference in Invitro Dry matter digestibility of about 7.4%, which is considered very high in terms of its impact on animal productivity and enteric methane emission has been observed (Subudhi et al., 2020). Crop breeding for improving nutritional quality of crop residues holds a potential for improving quality of crop, animal productivity, and reducing emission intensity. However, is it important to make sure that crop breeding for improvement of crop residue quality simultaneously increases grain yield, or at least does not decrease it. Otherwise such interventions will not work with African smallholder farmers who prioritize food than feed production (Adesogan et al., 2020).

### Improving nutritional status of animals

These strategies for improving nutritional status include ration balancing, supplementation with concentrates, and the use of additives to improve feed use efficiency and reduce enteric CH_4_ emissions.

#### Ration formulation.

In most small-scale production systems in Africa, animals are fed whatever is available to fill their rumen, without due consideration to the feed quality and animal requirement (Balehegn et al., 2020). Balancing the ration in dairy cows has been reported to reduce emission intensity of milk by 13.5%, and gross energy loss through CH_4_ by 10.3% (Garg et al., 2013). Balancing protein requirements reduces excess nitrogen excretion, thereby reducing N_2_O emissions through manure. Reducing crude protein in beef cattle diets from 13% to 11.5% reduced nitrogen emissions by 19% (Cole and Todd, 2009). However, reduction of dietary protein for dairy cattle must be closely managed to avoid negatively affecting milk production. Proper use of ration-formulation technology may require understanding and accurate quantification of the nutrient requirement of local breeds. The most commonly used nutrient requirement has been derived from experiments using Western (*Bos taurus*) breeds such as the Holstein-Friesian (Sauvant et al., 2017). However, SSA cattle are dominantly *Bos taurus taurus × Bos indicus* which have different requirements than western breeds (lower requirement for NE_L_ at maintenance (Oliveira, 2015). Therefore, there is a need for development of reference systems for local and cross-bred breeds.

#### Supplementation.

The widespread low livestock productivity in SSA is largely due to the nutritional inadequacy of rations. This can be addressed by providing supplementary nutrients and energy through adding or substituting low-quality feed with others that are greater in quality. In Ethiopia, replacing crop residues with legumes such as *Leucaena leucocephala* (Lam.) de Wit, *Moringa stenopetala* (Bak.f.) Cuf., *Sesbania sesban* (L.) *Merr., Cajanus cajan* (L.) Millsp., *Crotalaria juncea* L., and *Lablab purpureus* L. (Sweet) reduced enteric CH_4_ production by 8–26% (Berhanu et al., 2019). A simulation of corn replacing 20% roughage resulted in a 23% mean decrease in enteric CH_4_ emissions in three production systems in Ethiopia (Berhe et al., 2020). However, the reduction in CH_4_ emissions due to concentrate supplementation may be offset by increases in other non-enteric sources such as land conversion, processing, and transportation, when more grains are fed (Guo and Gifford, 2002; Peters et al., 2012). The main challenge with supplementation is the high cost of energy or nutrient-dense ingredients due to importation or competition for use in human diets. Increased and efficient domestic production of such ingredients is critically needed to reduce production costs and affordability by smallholder farmers.

### Adopting climate-smart livestock production systems

Climate-smart livestock interventions and strategies are applied to improve livestock productivity, especially, under resource-limited systems, while enhancing climate change adaptation, mitigation, and environmental resilience (Gaitán et al., 2016). Climate-smart farm-level integration of various components of the livestock system can reduce GHG emissions and emission intensities, both enteric and from manure, and increase carbon sequestration, ultimately contributing to a system-wide negative carbon balance (Ortiz-Gonzalo et al., 2017).

#### Agroforestry systems.

Carbon neutral feed production systems, such as silvopastoral and other agroforestry systems, can contribute to a reduction in GHG emissions from livestock through improved feed quality and contribution to carbon sequestration (Feliciano et al., 2018). For instance, incorporating crop fodder tree plantation increased topsoil carbon by 141% compared to continuous corn cropping (Mutuo et al., 2005).

#### Grazing and rangeland management.

Proper grazing management can increase biomass production and biodiversity (e.g., Savory and Butterfield, 1998). About 1.5 billion tons CO_2_ equivalent/year, an amount sufficient to offset all the emissions from livestock production, can be sequestered by improving management of grazing lands (IPCC, 2007). Furthermore, well-managed grasslands can store up to 260 tons of carbon per ha (FAO, 2007) as rangelands can store up to 30% of the world’s soil carbon, over and above the substantial amount of aboveground carbon stored in trees, bushes, shrubs, and grasses (Grace et al., 2006). Interventions like zero-grazing, moderate and rotational grazing can increase livestock productivity and carbon sequestration by grazing lands (Gebregergs et al., 2019). Additional research is needed to understand how to manage grasslands to promote carbon capture and which mechanisms can be used to encourage such management practices.

#### Improved forage-based systems.

Unlike feedlots, perennial, low-input fodder systems can generally sequester a significant amount of carbon. Of the overall mitigation potential in agriculture, 50–80% is attributed to sown forages (Peters et al., 2012). Cultivated forages in low-income countries contribute about 4% of the overall global agricultural GHG mitigation potential (Thornton and Herrero, 2010). The benefit of forage-based systems is pronounced with improved forages as they increase herbage biomass and livestock productivity (Paul et al., 2020). When forage technologies were integrated with food crops, soil loss was almost halved, soil organic carbon increased by 10%, and grain and stover yields by 60% and 33%, respectively (Paul et al., 2020).

#### Preserving natural ecosystems.

In terms of soil carbon sequestration potential, pasturelands had better soil carbon content than croplands and even forests by a factor of 59% and 10%, respectively (Guo and Gifford, 2002), though changes are dependent on biophysical factors and geography (Powers et al., 2011). Avoidance of grazing, cultivation and other disturbances of degraded grasslands in northern Ethiopia resulted in an increase of carbon stock by 187% compared to a freely grazed area (Balehegn et al., 2019). Zero grazing and using enclosures on degraded grazing lands have greatly improved soil and aboveground carbon sequestration potential in many areas of northern Ethiopia (Gebregergs et al., 2019). However, despite its potential, the contribution of pastoral ecosystems such as those in SSA in offsetting atmospheric greenhouse gases through carbon storage is seldom appreciated.

#### Other interventions.

Other interventions that can help minimize the environmental impacts of livestock in SSA include manure management, housing, improved water use efficiency, animal health, and genetics (Knapp et al., 2014). In Ethiopia, a simulation where 20% of manure burnt for fuel or deposited on rangelands was managed as solid manure reduced CH_4_ and N_2_O emission from manure by 18–36% depending on the type of livestock production systems (Berhe et al., 2020). Herd management strategies such as improving herd efficiency and health and genetics; heat abatement, fertility management, and facility design; reducing herd sizes to retain only productive and efficient animals; ensuring attainment of market size or weight earlier can reduce total GHG emission from livestock by up to 30% (Gerber et al., 2013). Culling large number of unproductive animals and replacing them with fewer but more productive animals would result in reduced GHG emissions and other negative environmental impacts of livestock in Africa. However, the technique is particularly opposed to the objectives of farmers in Africa who prefer to keep larger numbers of animals for noneconomic purposes (Forabosco et al., 2017).

### Socioeconomic sustainability of livestock

Greenhouse gas emissions are often used as a proxy for livestock contributions to climate change, but this is just a single indicator of sustainability. The contribution of livestock to GHG emissions has been overestimated (Steinfeld et al., 2006) leading to recommendations of reduced consumption of ASF for environmental and other reasons (Willet et al., 2019). Additional critically important indicators of sustainability include social, nutritional, and economic factors and livestock play vital roles in in all these areas in low- and middle-income countries, particularly in Africa. For instance, ASF consumption is vital for ameliorating the high stunting rates that prevail across the SSA, because the World Health Organization (2014) noted that ASF are the best nutrient-rich foods for infants aged 6–23 mo, and UNICEF (2019) noted that about 59% of children in the world do not get the nutrients they need from ASF. In fact, World Bank researchers noted that the GDP can be reduced by 9–10% on average in African and Asian countries in which the workforce is made up of people who experienced childhood stunting (Galasso et al., 2016). Consumption of ASF is therefore vital for physical and cognitive development in such countries because the poor, particularly in rural areas, lack access or cannot afford other nutrient-dense foods. Livestock provide draught power to a third of farmers in developing countries (Bruinsma, 2003). Manure is used as organic fertilizer in half of the world’s croplands and mostly in developing countries, as well as for fuel and for building (Bruinsma, 2003). Livestock are symbols of status, a means of income and insurance, and are vital for resilience and livelihoods of numerous people. For instance, in Niger and Burkina Faso, approximately 80% of the population is involved in livestock production (Molina-Flores et al., 2020) and on average, livestock contribute 40% to the agricultural GDP of developing countries. Further, livestock are often an important and only source of income for landless female farmers in developing countries, making it a critical component of achieving economic gender equality (Bravo-Baumann, 2000). Consequently, several of the UN sustainable development goals on zero hunger, education, gender equality, poverty alleviation, environmental health, etc., cannot be achieved without livestock and livestock products (Adesogan et al., 2020). Further, more than 70% of feed utilized by livestock in Africa is crop residues that would otherwise be burned and contribute to more emissions (FAO, 2014), making livestock indispensable for economic and environmental sustainability.

For the reasons described above, achieving socioeconomic sustainability in the livestock sector in the developing countries must require a proper understanding of the roles and challenges with livestock production in developing countries (Adesogan et al., 2020). A combination of various sustainable livestock intensification strategies such as herd management strategies, adoption of feed improvement technologies, grazing management strategies, and more environmentally resilient and carbon-neutral production systems such as silvopasture can help prevent or reduce negative environmental impacts of livestock, while at the same time enhancing their social, economic, and nutritional roles.

## Policies to Address Greenhouse Gas Emissions and Other Sustainability Issues

The policy environment for managing the environmental footprint of livestock in Africa is underdeveloped relative to the United States or Europe, with few countries implementing any deliberate policies. This is not surprising given that across Africa, the livestock sector has not been well supported by policy initiatives in general (AU-IBAR, 2016). We look at two areas: reducing total GHG production and intensity from livestock, and land governance as it affects livestock production.

### Greenhouse gas emissions from livestock

Policies to reduce GHG emissions from livestock production in Africa are largely aspirational goals. These goals are stated in Nationally Determined Contributions (NDCs) that nearly every African country committed to after the 2015 Paris Agreement. Many countries in Africa include agriculture as a sector for achieving reductions in GHG emissions intensities (Richards et al., 2015), and within agriculture, the livestock sector is usually the largest emitter. Consequently, the livestock sector is often targeted as means of achieving NDCs. Ethiopia estimates that it could reduce GHG emissions from livestock production by 49 Mt CO_2_e by 2030 as part of its widely heralded Climate Resilient Green Economy strategy (Federal Democratic Republic of Ethiopia (FDRE), 2011). However, in most cases, countries have set targets with little to no concrete strategies to achieve these targets. Ethiopia is one of the few exceptions, but even there, a lot of work remains (Kristjanson, 2020).

Implementation of strategies to achieve the targets remains a challenge because African countries need several kinds of resources. First, they need data and an institutional structure to establish a GHG inventory for livestock so that they can convincingly monitor progress. Reliable data for livestock are often missing. Kenya, for example, has not updated its livestock census in over a decade. Furthermore, agencies struggle to share data or to understand what is needed to establish a GHG inventory that meets international reporting standards (T.A.C., personal observation). In addition to data, countries need validated Monitoring, Reporting and Verification (MRV) systems in place where data can be integrated. To date progress for the livestock sector has been slow (Wilkes et al., 2020), though Kenya has recently released an inventory of GHG emission for the dairy sector between 1995 and 2017 (GoK, 2020)

Second, countries need investments to finance the interventions proposed to achieve their mitigation targets. Attracting this is also difficult. For example, in Kenya, a Nationally Appropriate Mitigation Action for the dairy sector was drafted several years ago, but the proposal has yet to find investment support (NAMA Investment Proposal, 2014). Rapid progress in low emissions development is expensive: governments need interventions to stimulate millions of farmers to implement specific practices to achieve their ambitious targets. Farmers, in turn, need access to information along with financial and other resource incentives to make the investments profitable if they are to adopt them (Ericksen and Crane, 2018).

Because achieving national mitigation targets implies substantial changes in producers’ practices, successful low-emission development needs to find the space where producers’ needs for profitability overlap with national GHG targets. However, simultaneously meeting GHG targets and producers’ livelihood needs requires low-emission development initiatives to anticipate and engage with questions of producers’ variable priorities and capacities, as well sectoral political economies. For example, recent research from southern Tanzania shows that heterogeneity in milk-producing households’ economic organization, priorities, and capacities has significant implications for their ability and motivation to adopt dairy intensification technologies (Kihoro et al., 2021). Within households, shifts toward dairy intensification can add to women’s labor burden and disenfranchise them from profits. This dynamic, which emerges from cultural norms relating to market engagement and household headship, leads to women resisting some low emission development (LED) practices (Tavenner and Crane, 2018).

These initial findings indicate risks that rural development goals relating to equitable economic opportunity can be at odds with environmental goals associated with low-emission development interventions, though research on the social distributional dimensions of low-emission development is vastly underdeveloped compared to environmental research. With low-emission development emerging as a paradigm for investment in African livestock sectors, there is an urgent need for more research that analyzes the synergies and tensions between producers’ diverse livelihood needs and national governments environmental commitments, keeping in mind the variability in cultural norms relating to livestock keeping and market engagement across Africa (see Weiler et al., 2014).

The tension between mitigation and adaptation priorities is another important issue in African climate change investments. While African development priorities overwhelmingly emphasize the adaptation and food security aspects of climate-smart agriculture, the international donor community has emphasized climate mitigation through reduction of GHG emission intensities in the livestock sector without paying concomitant attention to adaptation. Countries’ emphasis on adaptation and food security is driven by a combination of factors. First, across much of Africa agriculture is highly vulnerable to the impacts of climate change and a large percentage of Africans still have agrarian livelihoods that rely to some degree on livestock (Descheemaeker et al., 2016). Second, a significant degree of food and nutritional insecurity still exists across Africa (FAO, 2017). Between the high vulnerability of agricultural livelihoods and nutritional deficits, reducing GHG emissions intensities from livestock is simply not a central principle for guiding rural development policy. However, within the dairy sector, on-farm practices that reduce GHG emissions intensities are highly overlapping with practices that constitute intensification, though it is important to note that intensification practices should not be assumed to improve producers’ profits (Ericksen and Crane, 2018). All the same, dairy intensification is slowly occurring in many countries, driven primarily by commercial interests, but it is not necessarily being monitored for or motivated by the reduction of GHG emissions intensities.

### Land management

Land management is an extensive topic covering it in detail is beyond the scope of this paper. Although most land management policies do not directly aim at the “sustainability” of livestock systems (Ashley, 2019), they influence related practices. For example, rangelands in East Africa are central to extensive livestock production, but a policy environment that has failed to control the processes driving fragmentation of these landscapes has resulted in the deterioration of the rangelands along with the livestock and wildlife that depend upon that vegetation (Galvin et al., 2008). Re-establishing land use planning with community management is crucial to restoring the viability of these rangelands (N’ganga and Robinson, 2018). Such efforts are key to using these vast rangeland areas to sequester more carbon, a concept that has gained international attention (Milne et al., 2016).

## Conclusions

Livestock are vital sources of livelihoods and nutrition to millions of smallholder livestock producers in Africa. Currently making up a third of the global livestock population, livestock in Africa are growing both in numbers and in demand. Urbanization and income growth are driving increased investment in livestock. However, the growing livestock population in Africa also poses environmental problems such as overgrazing, land degradation, increased GHG emissions or production, bush encroachment, and desertification. Even at current numbers, livestock in Africa are important contributors to global agricultural GHG. With African livestock numbers poised to increase in the future, GHG emissions as well as other negative environmental impacts of herding will increase. Current technologies and strategies, however, provide opportunities to focus attention on increasing productivity per animal rather than livestock numbers thus reducing negative environmental impacts. Such interventions include improving feed quality by upgrading crop residues, concentrate supplementation, that effectively reduce enteric CH_4_ production and emission intensity while improving feed conversion efficiency and miscellaneous sustainable livestock intensification strategies that improve productivity while minimizing the negative environmental impact of livestock. Such strategies include manure management, animal breeding, grazing practices, and sustainable forage production or pastureland management practices such as intercropping, silvopastoral practices, etc. Perhaps more than the need for new research is that for awareness creation about best bet technologies and approaches for improving livestock production and sustainability and for sustained extension support to enhance the adoption and use of available technologies and approaches. Given that African countries are already critically affected by climate change as manifested by extreme weather variability and recurrent drought, strategies that provide synergetic opportunities for climate adaptation and mitigation are needed for resource-limited smallholder farmers. Implementation of successful adaptation and mitigation schemes, however, is costly to smallholder farmers, and therefore policy support towards providing financial and technical incentives is required.

## About the Authors

**Dr Mulubrhan Balehegn** is an associate professor of Livestock production and pastoralist development at Mekelle University, Ethiopia. He holds a PhD degree in Animal nutrition From the Norwegian Institute of Life Sciences and an MSc in Livestock Production and Pastoralist Development from Mekelle University. His research interest includes improving the quality and productivity of animal feeds towards improving the productivity and consumption of animal source foods in developing countries. He is currently working as a research coordinator for Bill and Melinda Gate’s Foundation-funded project at the University of Florida. Before his current position, Dr Mulubrhan has worked as a postdoc fellow at the United Nations International Ecosystem Management Partnership (UNEP-IEMP) and at the Research and Publication Office, Mekelle University.

**Prof. Adugna Tolera** is an animal scientist specialized in animal feeds and nutrition with over 30 yr of experience in teaching, research and outreach activities. His research and development work experience focuses on animal feeds and nutrition, livestock production systems, feeding and management of meat and dairy animals, nutrition-health interaction, sustainable intensification of mixed crop-livestock production, rangeland management, and climate change. He has supervised several PhD and MSc students and published extensively. He provides advisory services to different international and national organizations in the areas of livestock production, feed resources and feeding systems, feedlot management, feed resources conservation, rangeland management, and project evaluation.

**Dr Todd A. Crane** is a Senior Scientist in the Sustainable Livestock Systems program at the International Livestock Research Institute in Nairobi, Kenya. With over 15 yr of experience in East and West Africa, his work focuses on the social and institutional dynamics of climate change adaptation and low-emission development in agricultural and pastoral systems. In specific, his work examines the interactions between changing social and technical practices in agriculture—ranging from intrahousehold labor dynamics to political economy in value chains to policy structures and incentives—in order to promote more sustainable and socially equitable solutions for rural development in Africa.

**Dr Polly Ericksen** leads the research program on Sustainable Livestock Systems at the International Livestock Research Institute (ILRI). She has a PhD in Soil Science and an MSc in Economics from the University of Wisconsin-Madison, and a BS in History from Swarthmore College. She has over 25 yr of experience working on agricultural development, natural resource management, and global environmental change in developing countries. This includes experience working for the World Agroforestry Centre, Catholic Relief Services, a research fellowship at Columbia University’s International Research Institute for Climate and Society (IRI), and 5 yr at the University of Oxford working with the Global Environmental Change and Food Systems (GECAFS) initiative of the Earth Systems Science Partnership.

**Sarah Hunt** is a science writing consultant with training in environmental anthropology, ecology, and biology. Recent consulting work has incorporated livestock systems, food and nutrition, and plant disease emergence and detection. Her personal research interests are technology innovation and adoption with a particular interest in ecological engineering and green technologies

**Dr Ermias Kebreab** is Associate Dean and holds the Sesnon Endowed Chair in Sustainable Animal Agriculture at UC Davis. He conducts research in nutrition modeling of biological systems and conducts experiments to reduce impact of animal agriculture on the environment, including the sustainable use of feed additives. He is contributing author to the 2019 IPCC update and chairs feed additive committee of Food and Agriculture Organization. He has authored over 200 peer-reviewed articles. He received several awards including AFIA Ruminant Nutrition Award. He received BSc from University of Asmara, Eritrea, MSc and PhD from University of Reading, UK.

**Dr Adgebola T. Adesogan** is the Director of the Feed the Future Innovation Lab for Livestock Systems and a Professor of Ruminant Nutrition at the University of Florida. He is currently the Chair of the Council of Feed the Future Innovation Lab Directors. His research interests include using animal-source foods to improve human health and nutrition, improving food and feed safety, quality, and preservation as well as devising sustainable strategies to optimize the performance, health, and welfare of livestock. Prior to his tenure at the University of Florida, he was an Assistant Professor of Animal Nutrition at the University of Wales, UK. He has served on the editorial boards of various animal science journals, mentored many PhD and masters’ students, and authored or coauthored more than 200 scientific publications. He has earned many honors, including awards from the American Dairy Science Association, the Faculty Commons, and the Florida Agricultural Experiment Station. In 2018, he was named International Educator of the Year by the University of Florida’s College of Agricultural and Life Sciences.
